# P86 1990–2021 trend in fatal burden from infections resistant to antibiotics across the UK background

**DOI:** 10.1093/jacamr/dlag102.092

**Published:** 2026-06-26

**Authors:** Gisela Robles Aguilar

**Affiliations:** University of Oxford, Oxford, UK

## Abstract

**Objectives:**

This analysis provides bacterial antimicrobial resistance (AMR) burden estimates for 1990–2021 across the UK with forecasts to 2050.

**Methods:**

The GBD 2021 Antimicrobial Resistance Collaborators recently published age-specific estimates of the number of deaths and DALYs attributable to and associated with bacterial AMR for 22 bacterial pathogens, 84 pathogen–drug combinations across 11 infectious syndromes between 1990 and 2021. We present estimates disaggregated by two counterfactuals: one assuming resistant infections were susceptible (AMR-attributable burden) and one assuming infections did not occur (AMR-associated burden). Our aim is two-fold, first to produce credible estimates despite the heterogeneity of available data, and to offer insights and point to critical opportunities for strengthening monitoring and policy response in that can better tackle the current burden of AMR in the UK.

**Results:**

The number of deaths due to sepsis increased between 1990 and 2021 across the UK, especially when we consider COVID-related deaths between 2020 and 2021. Infectious-related deaths increased across the population aged 70 years and older. Lower respiratory infections (LRI) caused by resistant bacteria was the syndrome related to the largest number of deaths, which were primarily diagnosed and treated in hospital settings. Other infection-related syndromes frequently associated with AMR mortality include bloodstream infections and peritoneal/intra-abdominal infections. *Staphylococcus aureus* was the bacterial pathogen most commonly linked with deaths associated with antimicrobial resistance during the study period, with MRSA being the pathogen-drug combination with the largest number of attributable deaths in the UK. *Escherichia coli*, *Klebsiella pneumoniae, Streptococcus pneumoniae*, and *Pseudomonas aeruginosa* resistant to carbapenems were also frequently linked with deaths associated with antimicrobial resistance. Given the health loss arising from AMR, these estimates may also contribute to inform interventions to prevent and mitigate the increasing risk of AMR infections.

**Conclusions:**

Given the health loss arising from AMR, timely estimates on the burden of AMR contribute to inform interventions to prevent and mitigate the increasing fatal burden that AMR poses in the UK.

